# Structural basis of glycan specificity of P[19] VP8*: Implications for rotavirus zoonosis and evolution

**DOI:** 10.1371/journal.ppat.1006707

**Published:** 2017-11-14

**Authors:** Yang Liu, Shenyuan Xu, Andrew L. Woodruff, Ming Xia, Ming Tan, Michael A. Kennedy, Xi Jiang

**Affiliations:** 1 Tianjin Key Laboratory of Molecular Nuclear Medicine, Institute of Radiation Medicine, Chinese Academy of Medical Sciences and Peking Union Medical College, Tianjin, China; 2 Division of Infectious Diseases, Cincinnati Children’s Hospital Medical Center, Cincinnati, OH, United States of America; 3 Department of Chemistry and Biochemistry, Miami University, Oxford, OH, United States of America; 4 University of Cincinnati College of Medicine, Cincinnati, OH, United States of America; University of California at Los Angeles, UNITED STATES

## Abstract

Recognition of specific cell surface glycans, mediated by the VP8* domain of the spike protein VP4, is the essential first step in rotavirus (RV) infection. Due to lack of direct structural information of virus-ligand interactions, the molecular basis of ligand-controlled host ranges of the major human RVs (P[8] and P[4]) in P[II] genogroup remains unknown. Here, through characterization of a minor P[II] RV (P[19]) that can infect both animals (pigs) and humans, we made an important advance to fill this knowledge gap by solving the crystal structures of the P[19] VP8* in complex with its ligands. Our data showed that P[19] RVs use a novel binding site that differs from the known ones of other genotypes/genogroups. This binding site is capable of interacting with two types of glycans, the mucin core and type 1 histo-blood group antigens (HBGAs) with a common GlcNAc as the central binding saccharide. The binding site is apparently shared by other P[II] RVs and possibly two genotypes (P[10] and P[12]) in P[I] as shown by their highly conserved GlcNAc-interacting residues. These data provide strong evidence of evolutionary connections among these human and animal RVs, pointing to a common ancestor in P[I] with a possible animal host origin. While the binding properties to GlcNAc-containing saccharides are maintained, changes in binding to additional residues, such as those in the polymorphic type 1 HBGAs may occur in the course of RV evolution, explaining the complex P[II] genogroup that mainly causes diseases in humans but also in some animals.

## Author summary

Rotaviruses (RVs) are diverse, infecting humans and/or animals. Significant advances in understanding ligand-associated RV host ranges have been made but how such host ligands drive RV evolution leading to the diverse genotypes/genogroups already identified remains unclear. In this study, through solving crystal structures of P[19] VP8*-ligand complexes with two different ligands, we demonstrated how genetic variations could configure a totally new ligand binding site leading to a distinct new evolutionary lineage. Sequence comparison also identified further changes of the binding site which may occur over the course of RV evolution leading to different P[II] genotypes infecting different populations, including some animal species widely seen everywhere around the world. The elucidation of the genetic and evolutionary relationships among all members of the P[II] lineage including the two genotypes in P[I] is highly significant for advancing our understanding of RV host ranges, disease burden and zoonosis of human diseases.

## Introduction

Rotaviruses (RVs) are a major cause of severe gastroenteritis in children under the age of 5, causing 200,000 deaths[1–3]and account for 2 million childhood hospital admissions with an estimated cost of over 1 billion US dollars per year[4, 5]. It has been shown that RV attachment to cell surface carbohydrates, mediated by the VP8* domain of the spike protein VP4, is a required first step of an effective infection [6–9]. RVs are genetically diverse. Based on the VP4 sequences, the group A RVs have been grouped into 40 genotypes (P[1]-P[40])[10, 11]. In a study based on the VP8* sequence, the different RV genotypes have been grouped into five genogroups (P[I]-P[V])[12]. Different genotypes/genogroups cause diseases in different populations and/or various animal species and each genotype and genogroup may have distinct glycan binding specificities responsible for their host ranges or tropism.

The previously identified “sialidase-sensitive” animal genotypes in the P[I] genogroup are the first examples exhibiting such genotype specificities that require interaction with terminal sialoglycans for cell entry [13–15]. However, most other genotypes in the five genogroups are found to be sialidase-insensitive and many of them have been found to recognize HBGAs [12, 16–19]. Human HBGAs are highly polymorphic containing the ABO, secretor (H) and Lewis families with wide distributions in the world populations and therefore may significantly affect RV epidemiology and disease burden. For example, the P[III] RVs that infect humans and many animal species have been found to recognize the type A HBGAs that are shared between humans and many animal species [12]. The P[11] genotype in P[IV] has been found to recognize the type 2 HBGAs precursors, which may be responsible for their age-specific host ranges in neonates and young infants due to the stepwise synthesis of HBGAs that is developmentally regulated [18, 19]. The P[11] RVs also commonly infect bovines which is believed to be due the occurrence of the specific HBGA precursors in these animals [20].

Significant advances of the molecular basis of ligand-controlled host ranges of RVs have been made following crystallographic studies of the RV VP8*s in complex with their genotype-specific ligands, including the sialic acid dependent animal RVs P[3] and P[7], the A antigen binding P[14] that infect both humans and animals, and P[11] that recognizes the type 2 precursor and commonly infects neonates, young infants and some animals[16, 20–22]. Interestingly, the VP8*s from all these animal and human RVs adopt a similar galectin-like fold, and interact with a specific glycan in the cleft region although the P[11] cleft is wider than in the other three RVs[20]. In addition, while the glycan binding site of P[11] VP8* has moved to span almost the entire length of the cleft, the P[14] and P[3]/P[7] RVs shared a common binding site located in one corner of the cleft region. These data are valuable for our understanding of RV host ranges, RV evolution and particularly zoonosis as many RVs infect both humans and animals.

Despite recent progress in elucidating VP8*/receptor interactions, the molecular basis of host ranges of the major human RVs (P[4], P[6] and P[8]) in the P[II] genogroup that are responsible for over 90% of human infections remains unknown due to lack of a conclusive data detailing the precise interactions of VP8* domains with host ligands as receptors for these human RVs[17, 23, 24]. Crystal structures of the native VP8* of the P[4] and P[8] RVs also showed a galectin-like fold with a similar wider cleft between the two β-shifts as that of P[11] RVs, which has led to a deduction of a similar cleft-glycan-binding site for P[4] and P[8] RVs[16, 20, 22, 25]. However, in our recent study of another P[II] RV (P[19]) that is genetically closely related with the other P[II] RVs, we found that P[19] VP8*s have a unique property of binding two types of glycans, the mucin core and type 1 histo-blood group antigens (HBGAs) and may use a binding site different from those described above[26], indicating that the P[II] RVs may be evolutionarily distantly related with other genogroups. To seek direct evidence on the possible shifted ligand binding site of P[19] VP8* and to explore the molecular basis how could a single binding site accommodate two structurally related but distinct glycan ligands, in this current study we resolved the crystal structures of the P[19] VP8* in complex with the two glycans. The structural data showed that the P[19] VP8* uses a completely new binding site that is different from the one of other RVs and this single binding site is able to interact with either type I HBGA pentasaccharide Lacto-N-fucopentaose I (LNFP I) or mucin core 2 glycan with a similar binding mode. Structure based-sequence comparisons also confirmed the conserved binding site of P[19] with other P[II] RVs. Unlike the other three P[II] RVs that mainly infect humans, the P[19] RVs are rarely found in humans but commonly infect animals (porcine). Thus, our study helps establish the genetic and evolutionary relationships among these human and animal RVs, which advanced our understanding of RV host ranges, disease burden, epidemiology, and zoonosis of human diseases.

## Results

### Verification of P[19] VP8* binding HBGAs and mucin core glycans

**Fig 1:**
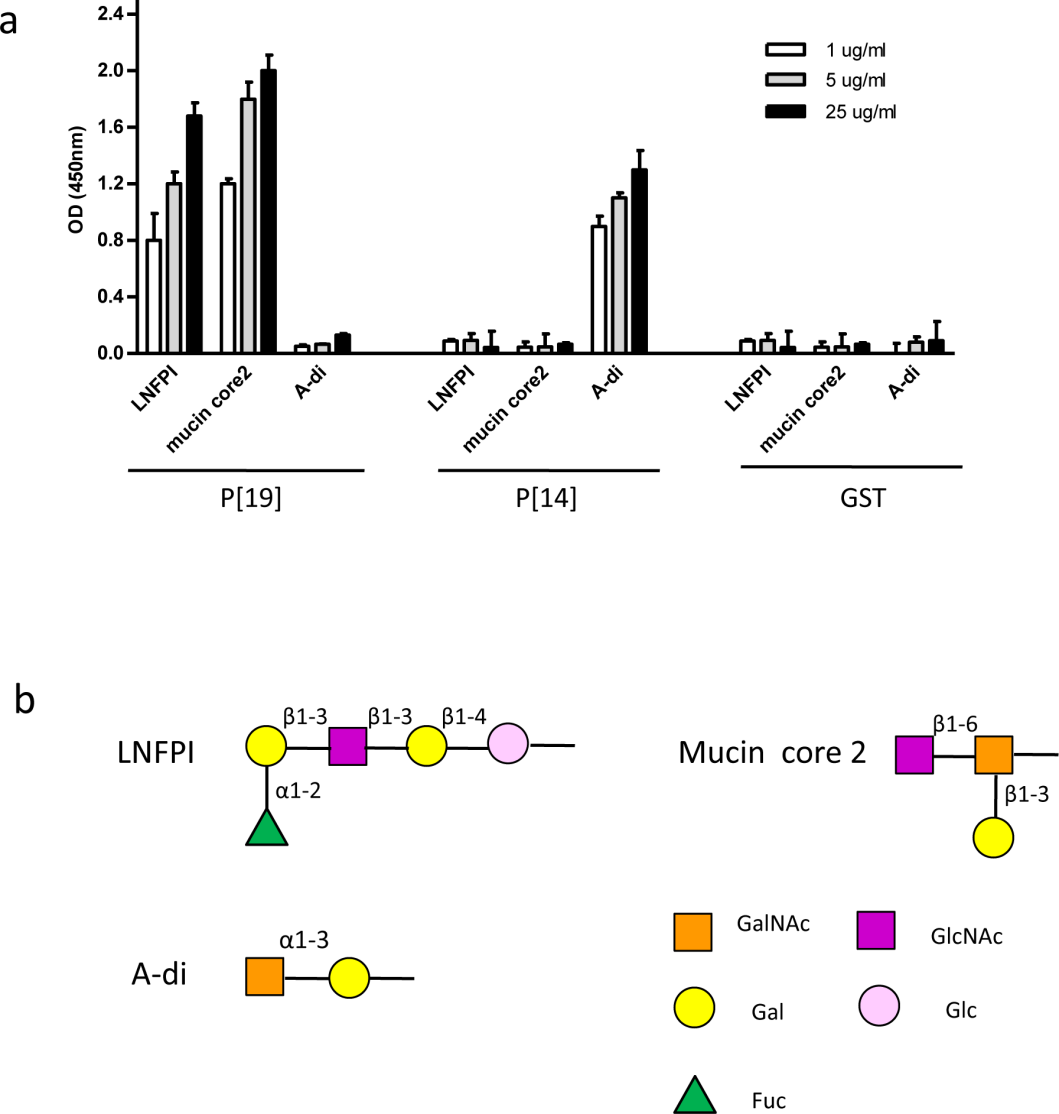
Binding of P[19] VP8* to LNFP I and mucin core 2. (a) A dose dependent binding of P[19] VP8* to the type I HBGA pentasaccharide Lacto-N-fucopentaose I (LNFP I) and mucin core 2. As a control, P[14] VP8* that specifically binds to the A-HBGA was included. Each condition was tested with three replicates. The error bars represent the standard deviation. (b) Schematic representation of the glycan structures. Gal, galactose; GalNAc, N-acetylgalactosamine; Glc, glucose; GlcNAc, N-acetylglucosamine; Fuc, fucose.

Previous glycan array studies have shown that the P[19] VP8* recognizes the mucin core glycans with a key GlcNAcβ1-6GalNAc motif and the type 1 HBGA precursor with inclusion of the internal Gal (Fucα1-2Galβ1-3GlcNAcβ1-3Gal)[26]. Prior to crystallographic studies, the binding specificity of P[19] VP8*was validated by ELISA using recombinant GST-tagged VP8* with the type 1 HBGA penta-saccharide LNFP I and mucin core 2 glycan (Fig 1). The binding of P[14] VP8* to A-type HBGAs was included as a positive control. Our results showed that the binding signals of P[19] VP8*exhibited typical dose-responses to both LNFP I and mucin core 2 glycans. As expected in the controls, the VP8* of a P[14] RV (P[III]) only recognized the A antigen but neither the LNFP I nor mucin core 2.

### P[19] VP8*-glycan complexes

The crystal structures of P[19] VP8* in complex with LNFP I and mucin core 2 were solved at 1.94 Å and 1.90 Å, respectively. The electron density was clear for both bound ligands, which allowed unambiguous assignment of the two ligands (S1 Fig). The secondary structural elements from N- to C- termini are designated as: βA (73–74), βB (80–85), βC (90–96), βD (102–108), βE (115–121), βF (124–130), βG (137–144), βH (152–159), βI (163–169), βJ (172–177), βK (184–189), βL (197–200), βM (204–208), and αA (212–221) (S2 Fig). In comparison with the previously solved P[19] VP8* native structure [27], ligand binding did not cause any significant conformational changes, with the root mean squared deviation (RMSD) for alpha carbons of the backbone atoms between the bound P[19] VP8* and the free VP8* being 0.531 Å (mucin core 2) and 0.505 Å (LNFP I), respectively. Interestingly, a structural rearrangement was noted among residues 87–90 in the B-C loop after P[19] VP8* bound to either of the glycans (S3 Fig), with the RMSD for alpha carbons of these residues between bound and free P[19] VP8* being 1.070 Å (mucin core 2)and 0.992 Å (LNFP I), respectively.

### P[19] VP8* exhibits a new glycan-binding site

**Fig 2:**
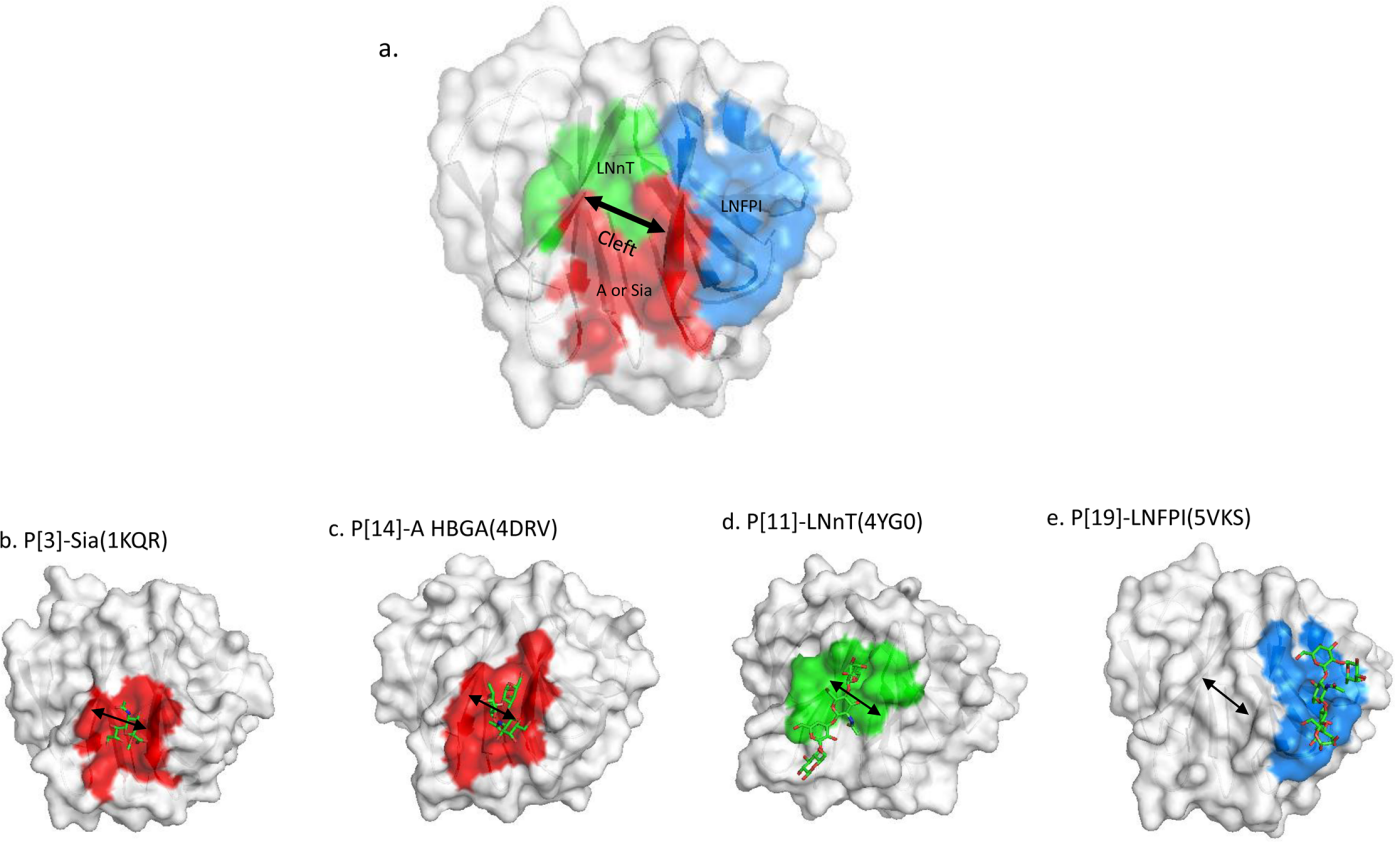
Comparison of ligand binding sites among P[3], P[14], P[11] and P[19] RVs with their specific ligands. (a) Schematics of known ligand binding sites based on the P[19] VP8* structures with different colors. The detailed ligand binding sites for each genotype are shown in (b)–(e). P[3] and P[14] shared a common binding site locating in one corner of the cleft (red color) to interact with the sialic acid or A antigen of the two genotypes, respectively (a)-(c). The ligand binding site of P[11] is also located in the cleft region (green color) while the cleft is wider and its ligand spread the entire cleft (d). P[19] characterized in our current study also has a wider cleft but the ligand binding site (blue color) is in a totally new location different from the other genotypes (e). Arrows indicate relative widths of cleft for individual genotypes.

**Fig 3:**
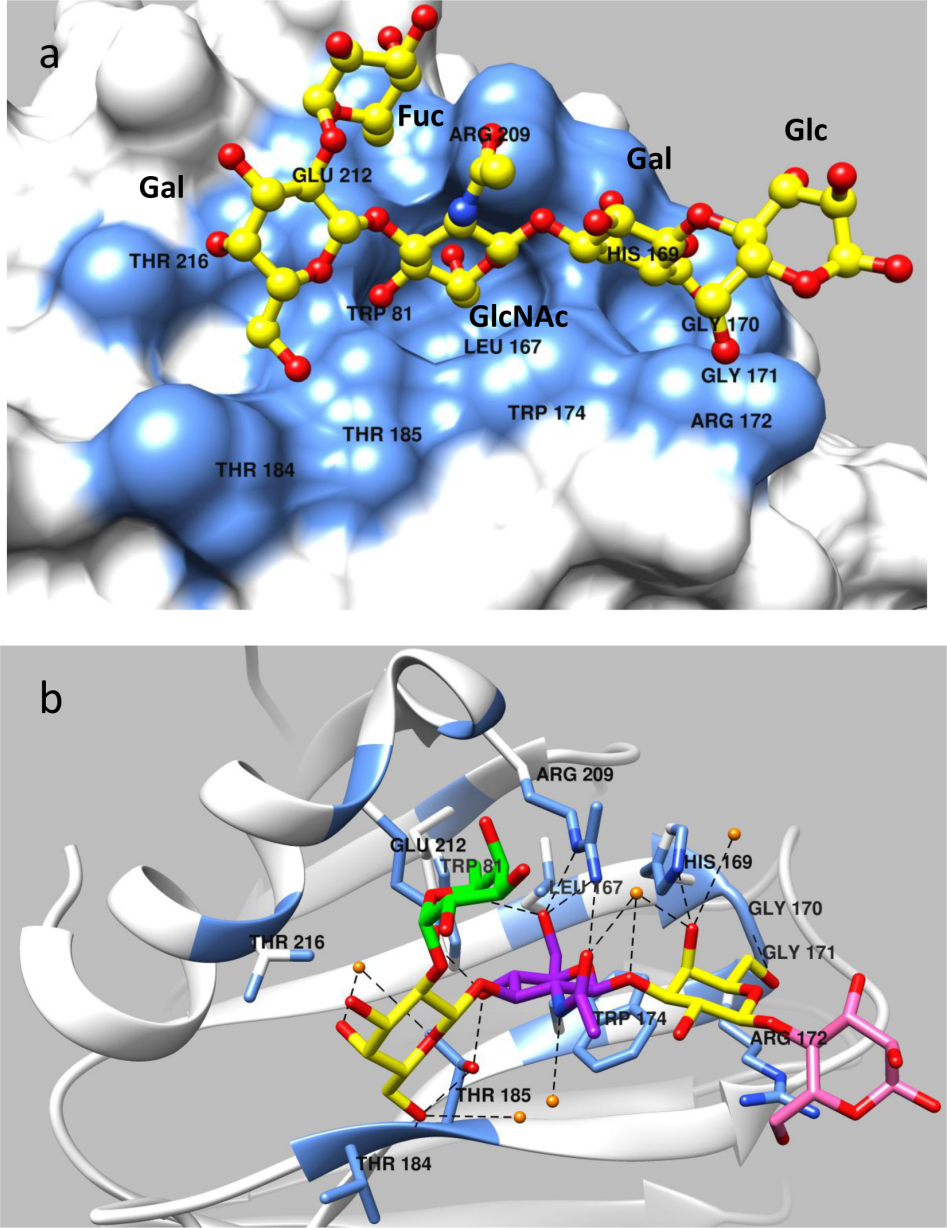
Structure of P[19] VP8* in complex with LNFP I. (a) Surface representation of P[19] VP8* (grey) with a bound LNFP I. The penta-saccharide LNFP I is shown in a ball-and-stick representation (yellow) with the nitrogen and the oxygen atoms being colored in blue and red, respectively. The amino acid residues in the P[19] VP8* which participate in hydrogen bonds and hydrophobic interactions with LNFP I are in blue color. The GlcNAc and an inner Gal moiety of the LNFP I (Fucα1-2Galβ1-3GlcNAcβ1-3Galβ1-4Glc) insert into two adjacent well-defined pocket in the P[19] VP8* structure. The Fuc projects out from the VP8* binding surface and does not have any direct contact with VP8*. (b) Network of hydrogen bond interactions (dashed lines) between the VP8* residues and LNFP I. The interaction residues are shown in stick model and the glycan is shown in stick representation with different colors, Fuc colored in green, Gal in yellow, GlcNAc in purple and Glc in pink. Participating water molecules are shown as small spheres (orange). Molecular interactions of LNFP I with P[19] VP8* were analyzed using LIGPLOT (see S4 Fig).

**Fig 4:**
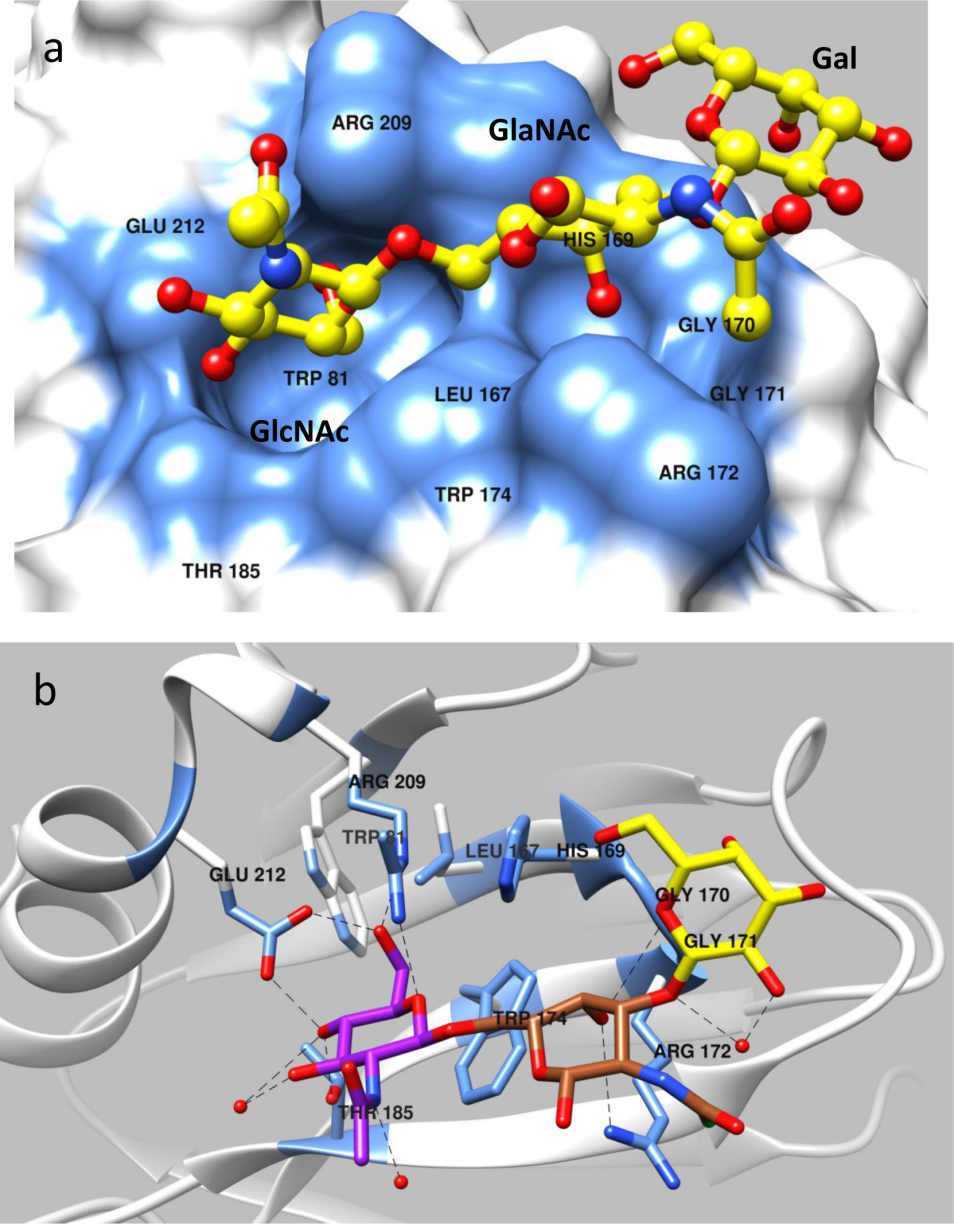
Structure of P[19] VP8* in complex with mucin core 2. (a) Surface representation of P[19] VP8* structure with bound mucin core 2 following the same coloring scheme as in Fig 2a. (b) Network of hydrogen bond interactions between the VP8* residues and mucin core 2 with the same coloring scheme as in Fig 2b. All the three sugar moieties of mucin core 2 are involved in interaction with the VP8*, with the GlcNAc and GalNAc of mucin core 2 (GlcNAcβ1-6GalNAcβ1-3Gal) binding to the two adjacent well-defined pocket on the surface of P[19] VP8*.

Similar to other known VP8* structures, P[19]VP8*s adopted a classical galectin-like fold with two twisted antiparallel β-sheets consisting of strands A, L, C, D, G, H and M, B, I, J, K, respectively (S2 Fig). The common shallow cleft between the two β-sheets where the glycan-binding sites of other known RVs are located (Fig 2a–2d) is wide in P[19] (Fig 2a and 2e) similar to the P[11] and P[8]/P[4] VP8*s but wider than the P[3]/P[7] and P[14] VP8*s. It was noted that the P[19] glycan binding site is located away from the cleft and thus represents a completely new glycan binding site, consistent with our previous observations based on NMR and mutagenesis studies [26]. This new glycan-binding site is composed of the carboxyl-terminal α-helix and the β sheet that composed of the βB, βI, βJ, and βK strands (Figs 2e and 3).The residues involved in the LNFP I interaction include W81, L167, H169, G170, G171, R172, W174, T184, T185, R209, E212, and T216, while those involved in mucin core 2 interactions are W81, L167, H169, G170, G171, R172, W174, T185, R209, and E212 (Fig 4).

### P[19] VP8* interacts with LNFP I and mucin core 2 through the same glycan-binding site

LNFP I binds P[19] VP8* with a network of hydrogen-bonding interactions and hydrophobic interactions (Fig 3). All four residues in the type 1 HBGA chain backbone, Galβ1-3GlcNAcβ1-3Galβ1-4Glc participated in the interaction, with the motif Galβ1-3GlcNAcβ1-3Gal playing a central role. The fucose in the penta-saccharide LNFP I, referred as Fuc-I, lies almost 90 degree relative to the plane formed by the rest of LNFP I residues and thus projects out from the VP8* binding surface without making direct contacts with VP8*. The galactose next to Fuc-I, referred as Gal-II, interacts with residues T184 and T216 via hydrophobic interactions, which is further stabilized by forming hydrogen-bonds with T185 and two water molecules. The GlcNAc at the third position (GlcNAc-III) inserts into the deep binding pocket formed by W81, L167, W174, T185, R209, and E212, forming hydrophobic interactions between the LNFP I backbone and residues W81, L167, W174, and E212, and forming hydrogen bonds between the LNFP I and the side chains of residues T185, R209, and E212 (Fig 3b). Two other water molecules help stabilize GlcNAc-III through hydrogen bonds with its acetyl moiety. The fourth saccharide (Gal-IV) makes contacts with G170, G171, and W174 through hydrophobic interactions and forms hydrogen bonds with H169, G170 and two water molecules. The fifth saccharide (Glc-5) interacts with R172 through hydrophobic effects.

**Fig 5:**
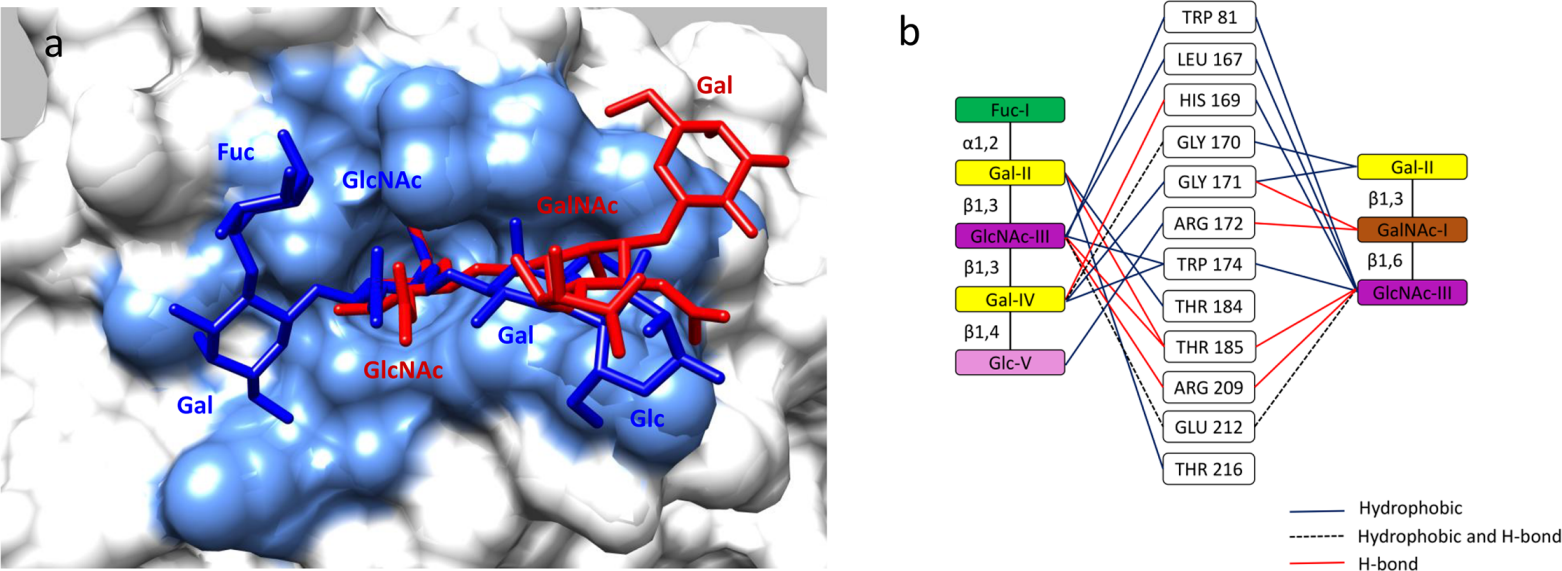
Structural comparison of P[19] VP8* in complex with LNFP I and mucin core 2. (a) Superimposition of P[19] VP8* complexes with LNFP I (dark blue) and mucin core 2 (red). Amino acid residues of the P[19] VP8* which participate in interactions with the two glycans are indicated in light blue. (b) Schematic interaction diagram of P[19] VP8* with the two glycans. Ten amino acids interacted with both glycans with two more (T184 and T21) interacted with LNFP I.

P[19] VP8* binds mucin core 2 using the same binding site (Fig 4) through an almost identical conformation as that of bind LNFP I, with a RMSD 0.118 Å for the alpha carbon backbone atoms. Two amino acids, T184 and T216, which are involved in binding to LNFP I, did not participate in binding to the shorter mucin core 2 trisaccharide (Fig 5). The GalNAc (GalNAc-I) forms hydrogen bonds with G171 and R172, while the Gal (Gal-II) makes hydrophobic interactions with G170 and G171. The amino acid residue of threonine that links to GalNAc-I pointed into the solvent. Interestingly, the GlcNAc (GlcNAc-III) inserted into the same deep binding pocket that binds the GlcNAc-III of LNFP I (Fig 3). It was noted that H169 contributed to the mucin core 2 binding interaction with GlcNAc-III, but this interaction was not seen with GlcNAc of LNFP I (Fig 5). The GlcNAc-III was further stabilized by two amino acids (T185, R209) through hydrogen bonding interactions. The superimposition of LNFP I and mucin core 2 in interaction with P[19] VP8* and the schematic interacting diagram was shown in Fig 5.

### P[II] RV replication could be inhibited by the two ligands

**Fig 6:**
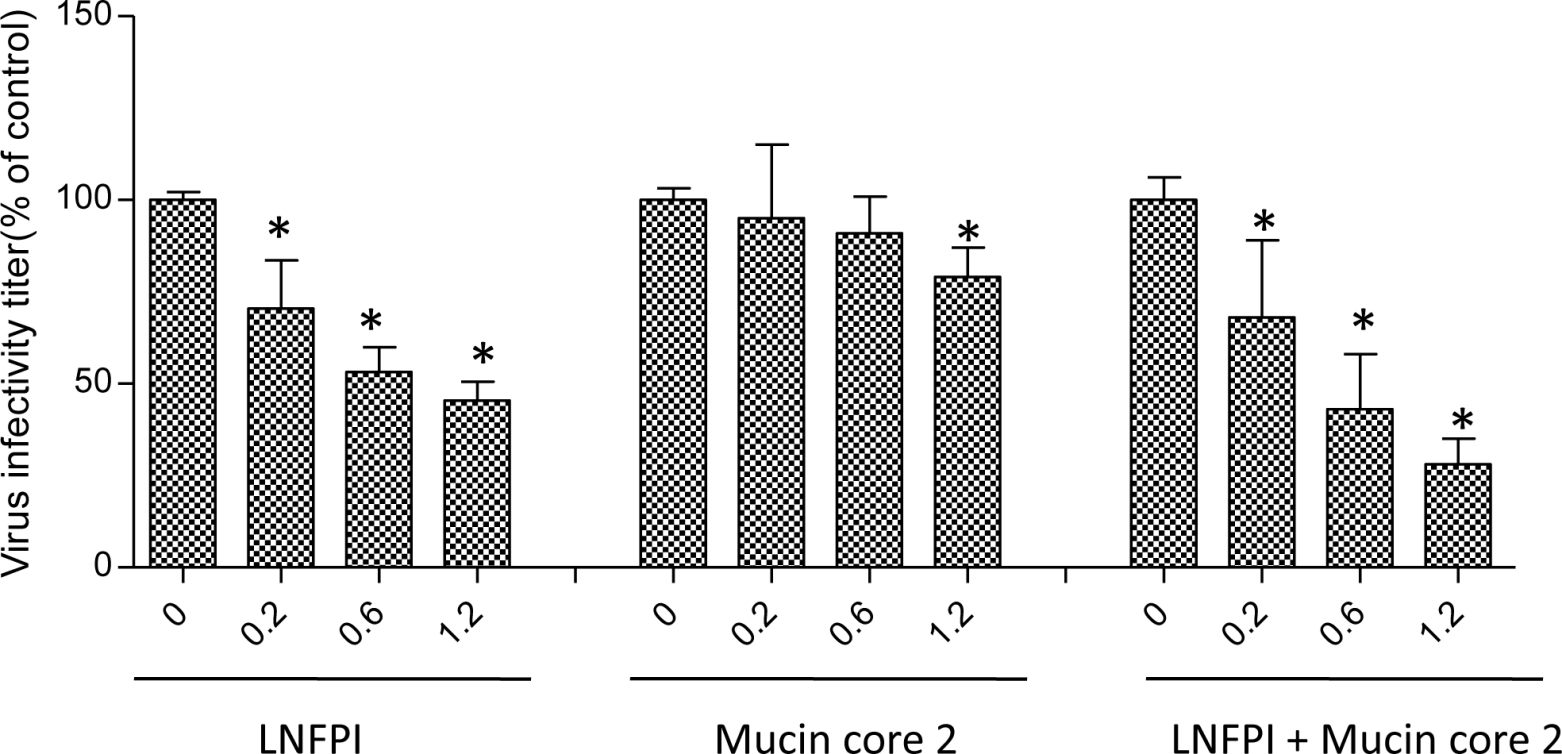
Inhibition of P[19] RV replication in cell culture by LNFP I and mucin core 2. LNFP I shows significant dose-dependent inhibition. Higher doses are required for mucin core 2 and adding of both LNFP I and mucin core 2 resulted in the highest inhibition. Error bars represent standard errors from triplicate repeats and the experiment was repeated once. The statistical significance was calculated by ANOVA, the asterisk refers to statistical difference <0.05.

To examine the biological significance of reactive glycan ligands to P[19] RV function, viral replication inhibition assays were performed on a human P[19] RV. P[19] RV titers were significantly reduced following incubation of the viruses with combination of both LNFP I and mucin core 2 (Fig 6).

### Structural conservation and variation of P[19] binding interface with other RV genotypes and genogroups

**Fig 7:**
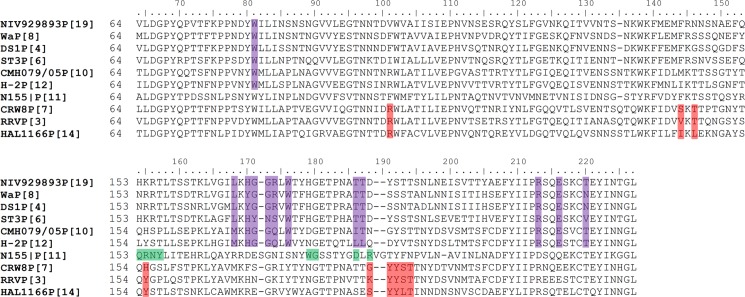
Structure-based sequence alignment of VP8*s. The VP8* sequences of P[19] and other P[II] (P[4], P[6] and P[8]) and two P[I] RVs (P[10] and P[12]) are included and the amino acids corresponding to the binding site of P[19] VP8* are highlighted in purple. The VP8* sequences of other RVs with known binding sites are also included. The amino acids of their binding sites are highlighted in red for P[3] and P[7] that recognize the sialic acids and P[14] that recognizes the A-HBGAs. The amino acids of the binding site of P[11] RVs are highlighted in green that recognizes the poly-linker type 2 HBGA precursors.

Structural-based sequence alignment of P[4], P[6], P[8] and P[19] among P[II] RVs showed significant amino acid conservation on the newly identified ligand binding interface (Fig 7). Residues W81, L167, W174, T184, T185, R209, and E212 are identical among all four P[II] genotypes based on representative sequences from each genotype, while there are slight difference at residues 169, 170, 171, 172, and 216. For example, the H169 in P[6] and P[19] is changed to Y169 in P[4] and P[8], and the R172 in P[4], P[8] and P[19] is changed to S172 in P[6]. As LNFP I and mucin core 2 share the same binding interface in P[19] VP8*, we use LNFP I as a representative to investigate whether P[4] and P[8] could accommodate the ligand in the newly found glycan-binding site as their native crystal structures are available.

**Fig 8:**
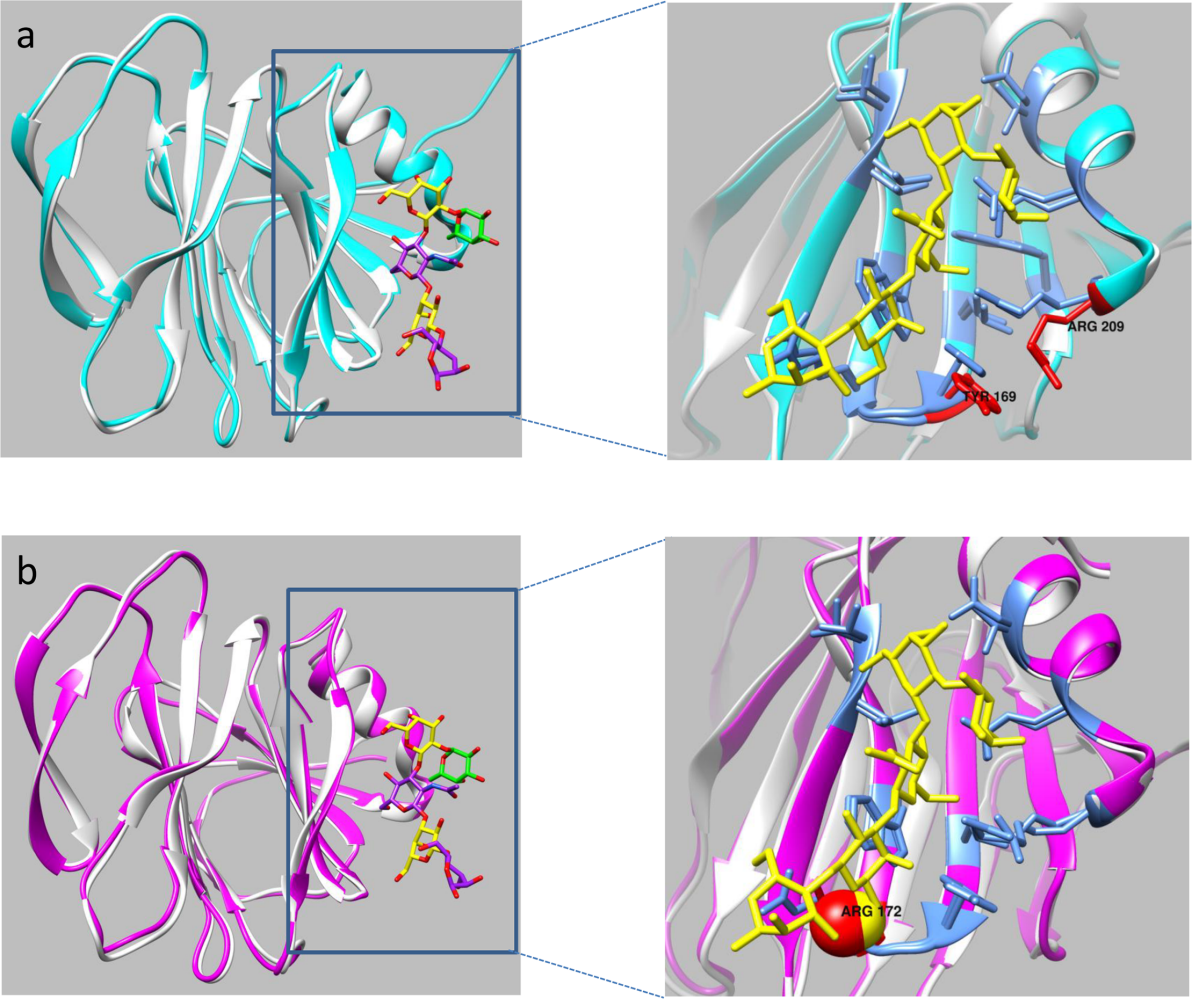
Superimposition of LNFP I-bound P[19] VP8* with apo VP8*s of P[4] and P[8] RVs. (a) The VP8* structure of P[4] (PDB ID: 2AEN) (cyan) and P[19] VP8* (grey) with LNFP I as colored sticks. The zoomed-in inset panel at the right shows a detailed view of the LNFP I binding interface defined by the P[19]-LNFP I complex structure. The structural overlay highlights the differences in the side chain orientations between the P[19] VP8-LNFP I-bound structure and apo P[4] VP8 structure. Residues Y169 and R209 in the apo P[4] VP8* tilt away from the binding pocket, which may destabilize the interaction with LNFP I. (b) Superimposition of the VP8* of P[8] (PDB ID: 2DWR) (pink) and the LNFP I-bound P[19] VP8*(grey) with the zoomed-in inset at right showing the detailed view of the ligand binding site in the P[19]-LNFP I complex structure. The side chain of R172 in P[8] VP8* sterically clashes with the oxygen atom of the Gal-IV moiety of LNFP I, providing a possible explanation for lack of P[8] VP8* binding to LNFP I (26).

The overall structures of the LNFP I-bound P[19] VP8* domain and the apo P[4] VP8* domain (PDB: 2AEN) were very similar based on the backbone alpha carbon RMSD of 0.569 Å(Fig 8a). Superimposition of the P[4] VP8* apo structure onto the P[19] VP8* structure in its complex with LNFP I showed that the LNFP I fit almost perfectly onto the surface of the apo P[4] VP8* defined by the same residues observed in the binding interface of LNFP 1 to the P[19] VP8* (Fig 8a, inset). The only notable differences were that the sidechains of Y169 and R209 in the P[4] VP8* tilted away from the LNFP 1 binding pocket, which may destabilize the interaction between LNFP I and P[4] VP8*. The LNFP I-bound P[19] VP8* also had a similar overall structure as the apo P[8] VP8* domain (PDB: 2DWR), with the RMSD of the alpha carbons of the backbone being equal to 0.493 Å (Fig 8b). Again, superimposition of the two structures indicated that the side chains of almost all of the residues involved in binding to LNFP I in the P[19] VP8* domain were also in position to interact with the LNFP 1 except that the side chain of R172 in the P[8] VP8* domain sterically clashed with the oxygen atom of the Gal-IV moiety of LNFP I in the superimposed structure, which likely is responsible for preventing P[8] VP8* from binding to LNFP I. These structural analyses suggested that P[4] and P[8] as well as P[6] (the crystal structure of P[6] remains unavailable) may all utilize the same ligand binding site identified in P[19], however, slight sequence and structural variations among the VP8* domains encoded within the different genotypes may be responsible for genotype-specific ligand-binding patterns and specificities of different genotypes.

## Discussion

Our crystallography studies clearly demonstrated that the P[19] RVs use a novel glycan binding site that is different compared to known binding sites of other RV genotypes and genogroups. This new binding site is capable of interacting with two structurally related but distinct glycans, the mucin core 2 and type 1 HBGA of LNFP I, using a common binding pocket and similar binding mode. Sequence comparisons showed high levels of amino acid conservation of the P[19] binding site with other P[II] genotypes (P[4], P[6] and P[8]) and two genotypes in P[I]. These data confirmed the new binding site of P[19] previously deduced from NMR and mutagenesis analyses[26], supporting the hypothesis that P[II] RVs are under strong selection of the host mucin core and/or type 1 HBGAs as common traits. However, given the complicated P[II] genogroup with multiple genotypes and variable host ranges among different human populations and/or some animal species, further defining the molecular basis of how these two structurally related host ligands drive RV evolution leading to such diverse P[II] genogroup is important.

Both mucin cores and HBGAs are O-linked glycans commonly seen in nature on the mucosal surfaces and cellular membranes of many mammals. These O-linked glycans are synthesized step-wisely by a group of glycosyltransferases, in which the GlcNAc-containing oligosaccharide motifs that are recognized by P[II] RVs serve as the starters or precursors (S5 Fig). For example, the type 1 HBGA precursor Galβ1-3GlcNAc can be extended to different A, B, H and Lewis HBGA products by adding one specific saccharide in each step. This process is developmentally regulated in the early lives of children [18, 20, 26], which may also occur in animals, leading to shared precursors, intermediates and/or full HBGA products in some animal species. Given the high sequence conservation constituting the GlcNAc-binding pocket among all P[II] RVs, we deduced that the P[II] genogroup may originate from an ancestor with a simple binding site recognizing these GlcNAc-containing motifs (GlcNAc-Gal) and circulating in one or a group of species that share such glycans. Such a binding site could further expand its receptor binding repertoire through genetic variations in the course of RV evolution by adapting to additional residues when encountering new hosts producing longer and more complicated HBGA products or along the course of RV-host co-evolution.

The above deduction assumes that, while the binding ability to the GlcNAc-containing moiety is maintained, extended interactions with additional saccharides could affect receptor ligand binding affinity and/or specificity, therefore potentially changing the binding outcomes. This assumption is supported by the observed variable binding patterns of the four P[II] RVs (P[4], P[6], P[8], and P[19]) to the tetra- (LNT), penta- (LNFP I) and hexa-saccharide (LNDFH I) of the type 1 HBGAs that either contain (LNDFH I) or do not contain (LNT and LNFP I) the Lewis epitopes[26]. The observed inability of P[4] and P[8] binding to the penta-saccharide LNFP I without the Lewis epitope is further supported by homology modeling of the native P[4] and P[8] VP8* structures in comparison with the LNFP I bound P[19] VP8* with an identification of binding clash for both P[4] and P[8] VP8* (Fig 8). In addition, the amino acids H169 and T216 of P[19] that are involved in interactions with the residue Gals next to GlcNAc have changed to Y169 and N216, which may also lead to different binding outcomes in the two genotypes. Finally, it was noticed that there is a slightly shifted orientation of the GlcNAc-containing motif inside the binding pocket between mucin core 2 and the type 1 HBGAs, leading to a significant orientation shift of the backbones between mucin core 2 and type 1 HBGA within the binding cleft (Fig 5a). This indicated a mechanism for how P[19] VP8* is able to achieve a broader binding specificity to the extended molecules of the two glycan types.

While the majority of P[I] RVs infect animals, most of the P[II] RVs infect humans. Thus, we deduced that the P[II] RV may come from P[I] with an animal host origin and were introduced to humans by adapting to the polymorphic human HBGAs, leading to different P[II] genotypes infecting different human populations and/or some animal species depending on their evolutionary stages. For example, the P[19] genotype may represent an early evolutionary stage after the ancestors of P[II] genogroup started adapting to human receptors but they may still retain the binding specificities to the backbones of the mucin core 2 and type 1 HBGAs. This may explain why the P[19] RVs are commonly found in animals (porcine) but rarely in humans. On the other hand, the P[4] and P[8] RVs are genetically closely related and both genotypes are well developed that recognize the much more matured HBGA product, the Lewis b (Le^b^) antigen that is widely distributed in humans, and together these two genotypes are responsible for ~90% of human RV infections worldwide. Furthermore, the P[6] RVs have been found to recognize the much less matured type 1 HBGA precursors, which is consistent with the fact that the P[6] RVs commonly infect neonates and young infants through recognizing the age-specific precursor glycans that commonly occur in the early lives of children [28, 29]. The P[6] RVs are also commonly found to infect porcine, likely through the type 1 HBGA precursor glycans that are share between humans and pigs. Thus, the elucidation of such genotype-specific host ranges controlled by the host HBGA makeups is important for understanding the disease burden and epidemiology therefore vaccine strategy against RVs based on the P type vaccine approach [30].

The findings of sequence conservation of P[10]/P[12] binding sites with that of the P[19] and similar glycan binding profiles between P[10] and P[19] [26] extends our understanding of P[II] evolution, in which these two P[I] RVs may represent even earlier ancestors than P[19] of the P[II] lineage. Both P[10] and P[12] are minor genotypes occasionally found in humans and bats and horses, respectively [31, 32], while the majority of other P[I] RVs were more commonly found to cause diseases in different animal species, indicating that the P[10]/P[12] RVs are unique in P[I] and should be evolutionarily grouped to the P[II] lineage. In fact, P[10]/P[12] were genetically closer to P[19], P[8]/P[4] and P[6] than the rest genotypes based on the full VP4 sequence phylogeny analyses [33, 34]. Thus, the P[10]/P[12] RVs are considered to be the earliest traceable ancestor of P[II] lineage. The reason of low abundance of these two genotypes in any species remains unknown.

In conclusion, P[II] RVs represent a unique evolutionary lineage starting from an ancestor in P[I] with a possible animal host origin. While the original binding specify to the mucin core and type 1 HBGA precursors is maintained, additional interactions with adjacent residues may have occurred during the evolution of the ancestor genotype as it adapted to human receptors. This led to the diversity of RV strains seen today, with some mainly infecting animals with others mainly infecting humans [26]. Since viruses in all group A RV genotypes and genogroups must be from a single ultimate common ancestor, the deduced evolutionary path of animal-to-human transition from P[I] to P[II], but not the other way around, may apply to other genotypes and genogroups. This deduction is important for RV classification and epidemiology, which may impact prevention and control strategies, such as vaccine design against RVs. For example, since the majority of the genotypes in P[I] exclusively infect animals, they may not be suitable for developing live human vaccine, because they may not be able to replicate in human guts due to the lack of proper receptor. This issue is urgent as the Jennerian approach is still widely used to develop live animal reassortant RV vaccines in many countries.

Our research still has certain limitations. For example, the deduced binding sites for the major human RVs P[4], P[6] and P[8] remain to be verified by co-crystallization studies of the VP8* in complex with their ligands. This task is challenging as our homology model data indicated that the free VP8* does not accept Lewis b, consistent with previous observations from several other groups [23, 24]. In addition, the precise saccharide sequences and structures recognized by the major P[II] RVs remain unknown. As the cleft where the P[19] binding site is located is long and fairly deep, it is likely to accommodate a long glycan extending to both sides of the GlcNAc-containing oligosaccharide motifs, and future studies to explore this issue by glycan arrays with more representative human glycan pools are necessary. Finally, it is noted that the new binding site of P[19] shifts toward the C terminus of VP8* and is located in the bottom of VP8*, which may need the support of VP5* to exhibit its true binding characteristics. This issue also needs to be studied.

## Materials and methods

### Expression and purification of VP8* proteins in *Escherichia coli*

The VP8* core fragments (amino acids 64 to 223) of the human RV P[19] with an N-terminal glutathione S-transferase (GST) tag was overexpressed in *Escherichia coli* BL21 (DE3) cells as previously described [12, 17]. Cells were grown in 1L Luria broth (LB) medium supplemented with 100 μg ml^-1^ ampicillin at 310 K. When the OD600 reached 0.8, 0.5 mM isopropyl-β-D-thiogalactopyranoside was added to the medium to induce protein expression. The cell pellet was harvested within 12 h after induction and re-suspended in the 30 ml phosphate-buffered saline (PBS) buffer (140 mM NaCl, 2.7 mM KCl, 10 mM Na_2_HPO_4_, 1.8 mM KH_2_PO_4_, pH 7.3). The cells were lysed by French press (Thermo Fisher Scientific, Waltham, MA), then the cell debris was removed by centrifugation at 12,000×g for 30 min. The supernatant of the bacterial lysate was loaded to a disposable column (Qiagen, Hilden, German) pre-packed with glutathione agarose (Thermo Fisher Scientific). After three washes with PBS buffer, the GST fusion protein of interest was eluted with elution buffer (10 mM reduced glutathione, 50 mM Tris-HCl, pH 8.0). The GST tag of the VP8* protein was removed using the thrombin (Thermo Fisher Scientific) after dialysis into the buffer (20 mM Tris-HCl, 50 mM NaCl, pH 8.0). The flow-through was collected after passing the mixture through glutathione agarose, and the purified protein was concentrated to about 10 mg ml^-1^ with an Amicon Ultra-10 (Millipore, Billerica, MA). The purity of the protein was judged using 15% SDS-PAGE stained with Coomassie Brilliant Blue.

### Enzyme-linked immunosorbent assay

The binding of purified VP8* to different glycans was confirmed by ELISA. Synthetic polyacrylamide polymer (PAA) conjugated oligomers were used to study their specificity as a ligand for P[19] RVs. Briefly, microtiter plates (Thermo Fisher Scientific) were coated with recombinant VP8* proteins (10 μg/ml) at 4°C overnight. After blocking with 5% nonfat cow milk, synthetic polyacrylamide polymer (PAA)-biotin conjugated oligomers (Glycotech, Gaithersburg, MD) were added at serial dilutions and incubated at 4°C overnight. The bound oligosaccharides were then detected using HRP-conjugated-streptavidin (Jackson Immuno Research Laboratories, West Grove, PA) and displayed using the TMB kit (Kierkegaard and Perry Laboratory, Gaithersburg, MD).

### Crystallization, soaking, data collection, and structure determination

The hanging-drop vapor-diffusion method was used for crystallizing human RV P[19] VP8* protein and co-crystallizing VP8* complexed with LNFP I and mucin core 2. Crystals were obtained from drops where 1 μl purified P[19] VP8* was mixed with 1 μl of the reservoir buffer: 0.5 M ammonium sulfate, 0.1 M sodium citrate tribasic dihydrate pH 5.6, 1.0 M lithium sulfate monohydrate. The sugar LNFP I (Dextra, Reading, UK) and threonine linked mucin core 2 (kindly provided by James C. Paulson at the Scripps Research Institute) were prepared at 60 mM and 100 mM, respectively, in PBS with 10% glycerol and soaked into the crystals. Diffraction data were collected at the Advanced Photon Source (APS) beamline 31-ID-D, Argonne National Laboratory, in Chicago, Illinois. A total of 360 images were collected using 0.5° oscillation during 20 s exposures. The images were integrated with MOSFLM [35], and scaled with SCALA [36]. Molecular replacement was performed with PHASER [37] using the coordinates of chain A from 5GJ6 [27] as the search model. Iterative model building was manually carried out in COOT [38], and refinements using 5% of reflection in Free-R set were carried out in REFMAC [39] implemented in the CCP4 suite [40]. The structure quality was assessed using Mol Probity [41]. Final model and scaled reflection data were deposited at the Protein Data Bank (PDB ID 5VKS and 5VKI). Processing and refinement statistics for the final model are presented in Table 1. The visualization and investigation of the final model was analyzed using Chimera [42].

**Table 1:** Diffraction data collection and refinement statistics.

	P[19]VP8*-LNFP I	P[19]VP8*-mucin core 2
**Crystal parameters**		
Space group	I 41	I 41
Unit cell parameters		
a; b; c (Å)	115.63, 115.63, 101.83	115.86, 115.86, 101.76
α; β; γ (°)	90.00, 90.00, 90.00	90.00, 90.00, 90.00
**Data Collection**		
Wavelength (Å)	0.97931	0.97931
R_merge_	0.158 (0.741)^a^	0.182 (0.794)
Resolution (Å)	1.94 (1.94–2.05)	1.90 (1.90–2.00)
Unique reflections	49564	52895
Mean [(I)/σ(I)]	8.20 (2.70)	7.20 (2.40)
Completeness	99.90 (100.00)	100.00 (100.00)
Multiplicity	7.60 (7.50)	7.60 (7.50)
**Refinement**		
Resolution (Å)	81.76–1.94	81.93–1.90
R-work	0.20117	0.22084
R-Free	0.22959	0.24050
Number of protein atoms	2562	2562
Number of amino acid residues	320	320
number of Ligands	2	2
number of Water molecules	198	202
Mean B-values		
Protein	25.975	26.793
Ligands	39.903	45.877
Solvent	33.261	34.196
RMS deviation		
Bond lengths (Å)	0.0125	0.0131
Bond angles (°)	1.4385	1.5122
Ramachandran statistics (%)		
Preferred regions	95.57	95.57
Allowed regions	4.43	4.43
Outliers	0	0

^a^ Values in parentheses are for highest-resolution shell.

### Virus propagation, inhibition and infectivity assay

The P[19] RVs (strain 210) which was cell-culture adapted by multiple blind passages on the MA104 cells (passage 4–6) were then used for inhibition assays with the oligosaccharides LNFP I and mucin core 2 using procedures described previously[26]. The P[19] RVs at 300 fluorescent forming units (FFU)/10 μl) were pre-incubated with different inhibition reagents for 30 min. After rinsing twice with serum-free DMEM and chilling of all reagents and the 24-well plates on ice, duplicated wells of confluent MA104 monolayers were inoculated with the virus-oligosaccharide on ice with continuous rocker platform agitation for 1 h. The inoculum was then removed and the cells were washed twice with ice-cold serum-free DMEM. The plates were then placed back in the 37°C incubator for 18 to 20 h prior to quantification of infected cells by immunofluorescence with a rabbit anti-rotavirus antibody followed by a FITC-labeled goat anti-rabbit secondary antibody.

## Supporting information

S1 Fig The 2F0_FC electron density maps (blue) of the glycan binding site of P[19] VP8* with LNFP I (a) and mucin core 2 (b).
(TIF)

S2 Fig Ribbon representation of P[19] VP8* with individual strands alphabetically labeled with letters. (TIF)

S3 Fig Superimposition of free P[19] VP8* (grey) with VP8*-mucin core 2 (cyan, a) and VP8*-LNFP I (blue, b) complexes, respectively. (TIF)

S4 Fig Detailed VP8* ligand interactions as determined using LIGPLOT. (a) P[19] VP8* in interaction with LNFP I. (b) P[19] VP8* in interaction with mucin core 2. All the amino acid residues and saccharide moieties involved in the interactions are labeled. Hydrogen bond interactions are shown as green dashed lines between the respective donor and acceptor atoms along with the bond distance. The van der Walls contacts are indicated by an arc with spokes radiating towards the ligand atoms they contact.
(TIF)

S5 Fig Synthetic pathway of the type 1 histo-blood group antigens (a) and the basic structures of mucin cores (b).
(TIF)
