# Supplementary figures and images for: Structural basis of glycan specificity of P[19] VP8*: Implications for rotavirus zoonosis and evolution
Source: PLoS Pathog. 2017 Nov 14;13(11):e1006707. doi: 10.1371/journal.ppat.1006707 (PMC5705156; doi:10.1371/journal.ppat.1006707)

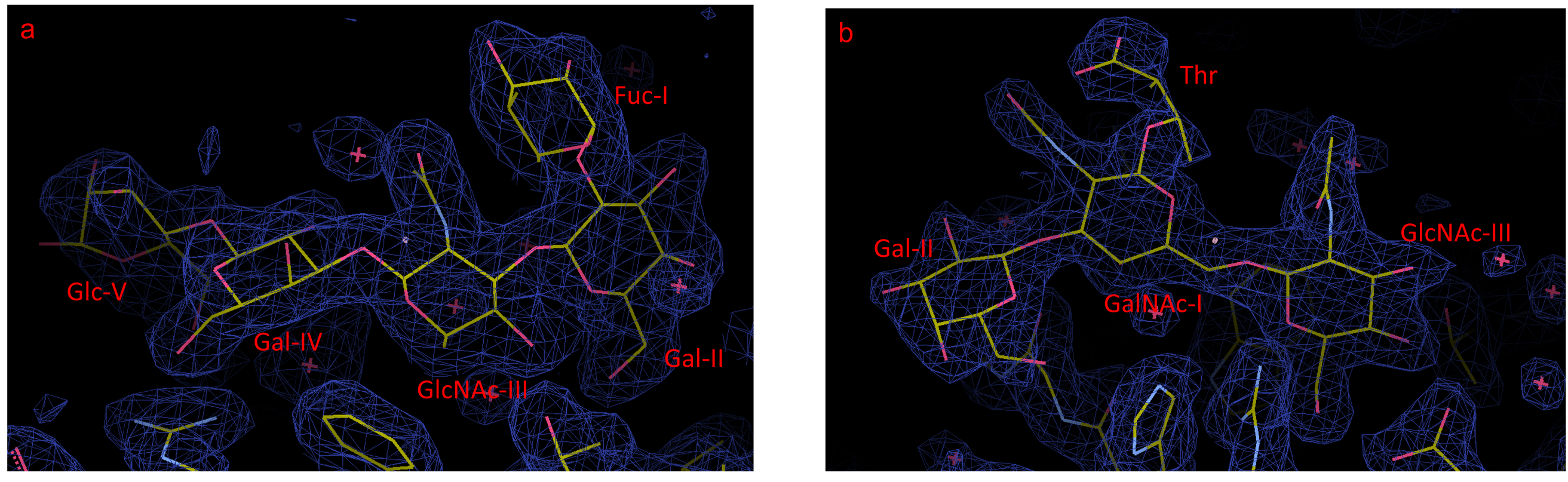

Supplement: S1 Fig — The 2F0_FC electron density maps (blue) of the glycan binding site of P[19] VP8* with LNFP I (a) and mucin core 2 (b). (TIF) [file ppat.1006707.s001.tif]

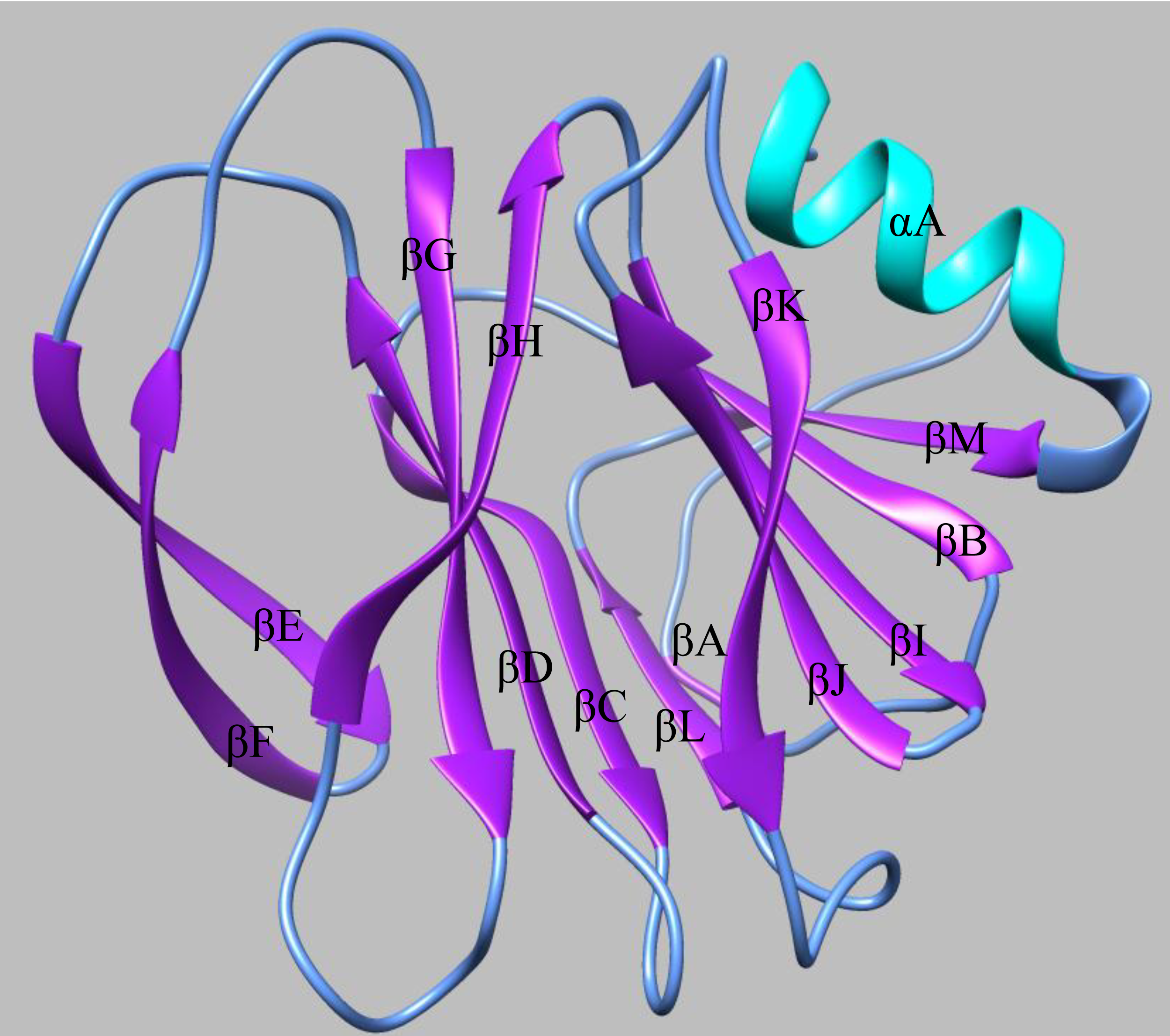

Supplement: S2 Fig — (TIF) [file ppat.1006707.s002.tif]

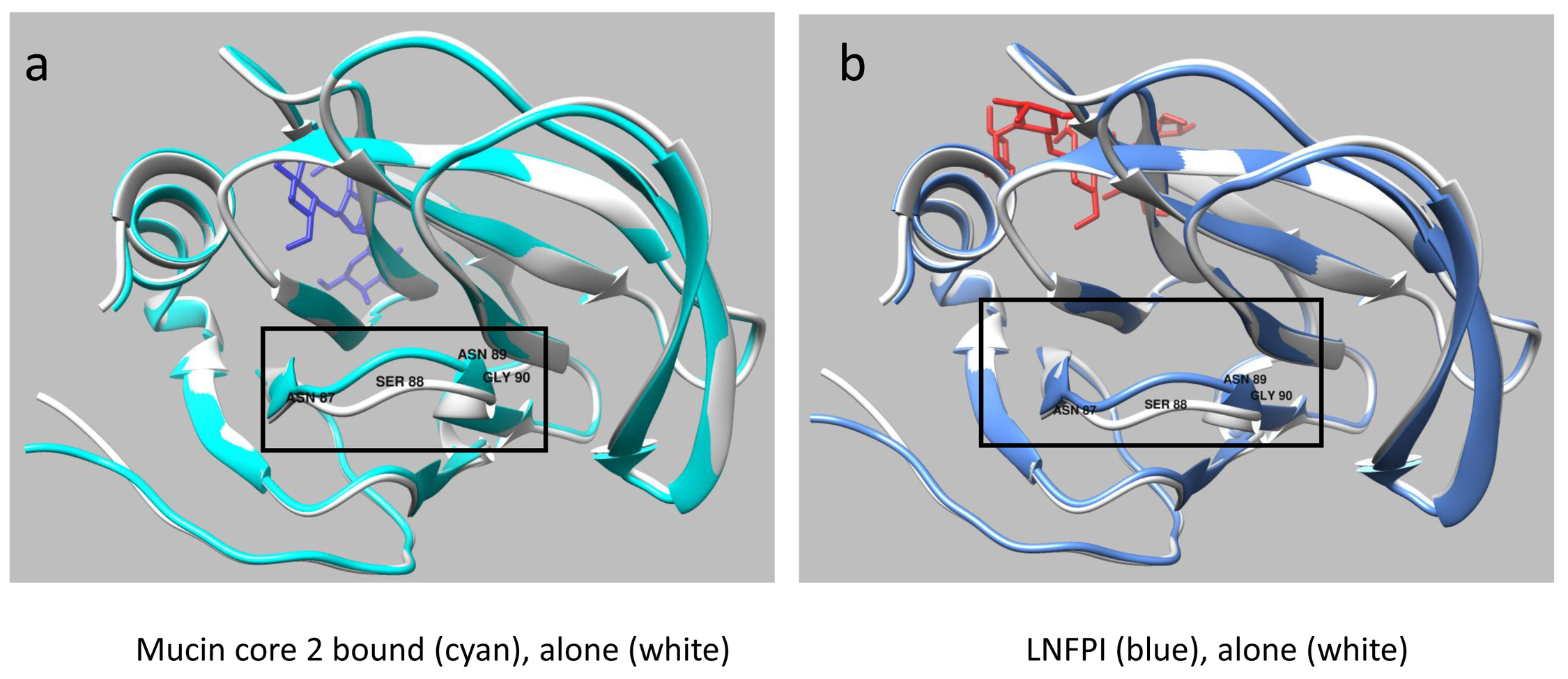

Supplement: S3 Fig — (TIF) [file ppat.1006707.s003.tif]

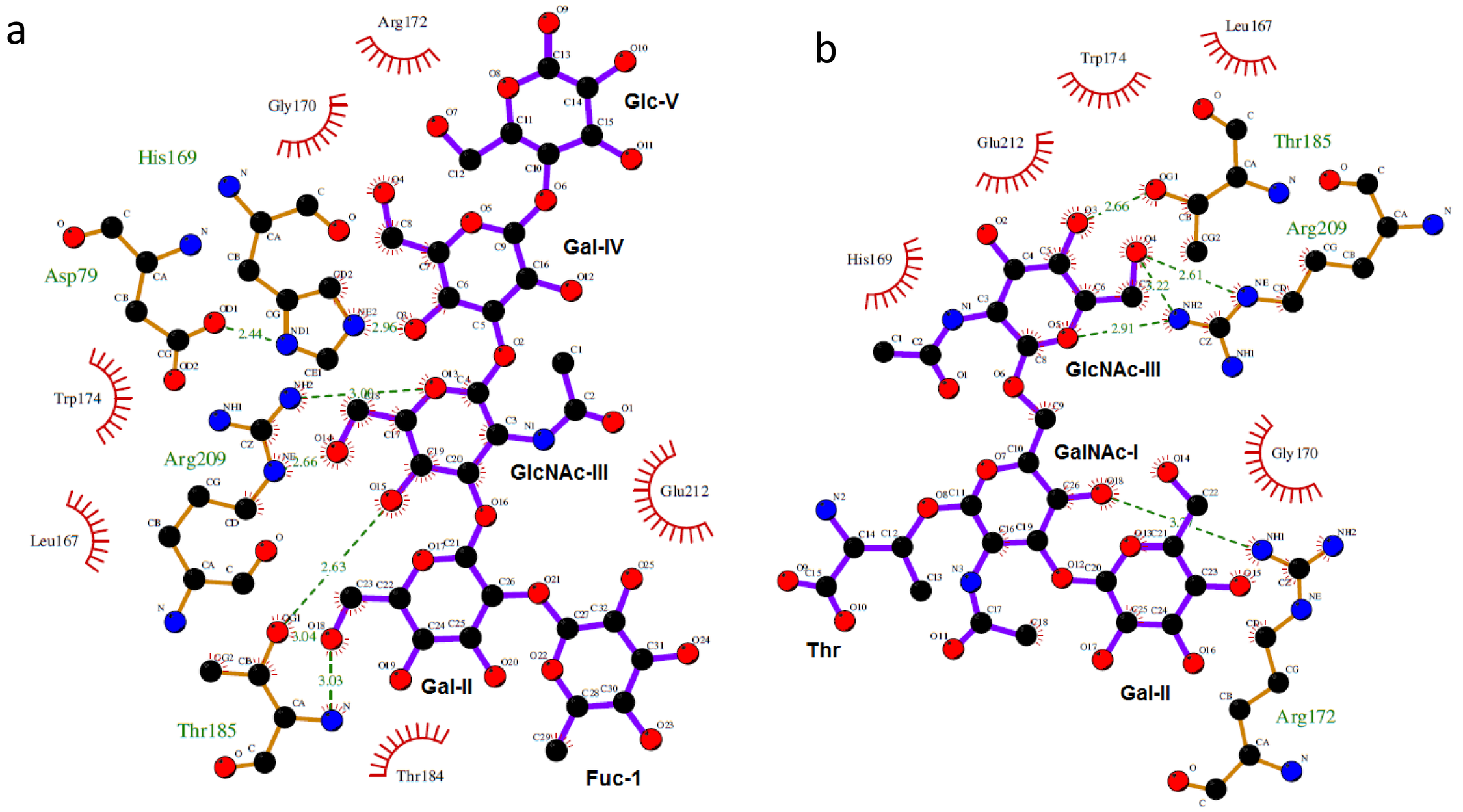

Supplement: S4 Fig — (a) P[19] VP8* in interaction with LNFP I. (b) P[19] VP8* in interaction with mucin core 2. All the amino acid residues and saccharide moieties involved in the interactions are labeled. Hydrogen bond interactions are shown as green dashed lines between the respective donor and acceptor atoms along with the bond distance. The van der Walls contacts are indicated by an arc with spokes radiating towards the ligand atoms they contact. (TIF) [file ppat.1006707.s004.tif]

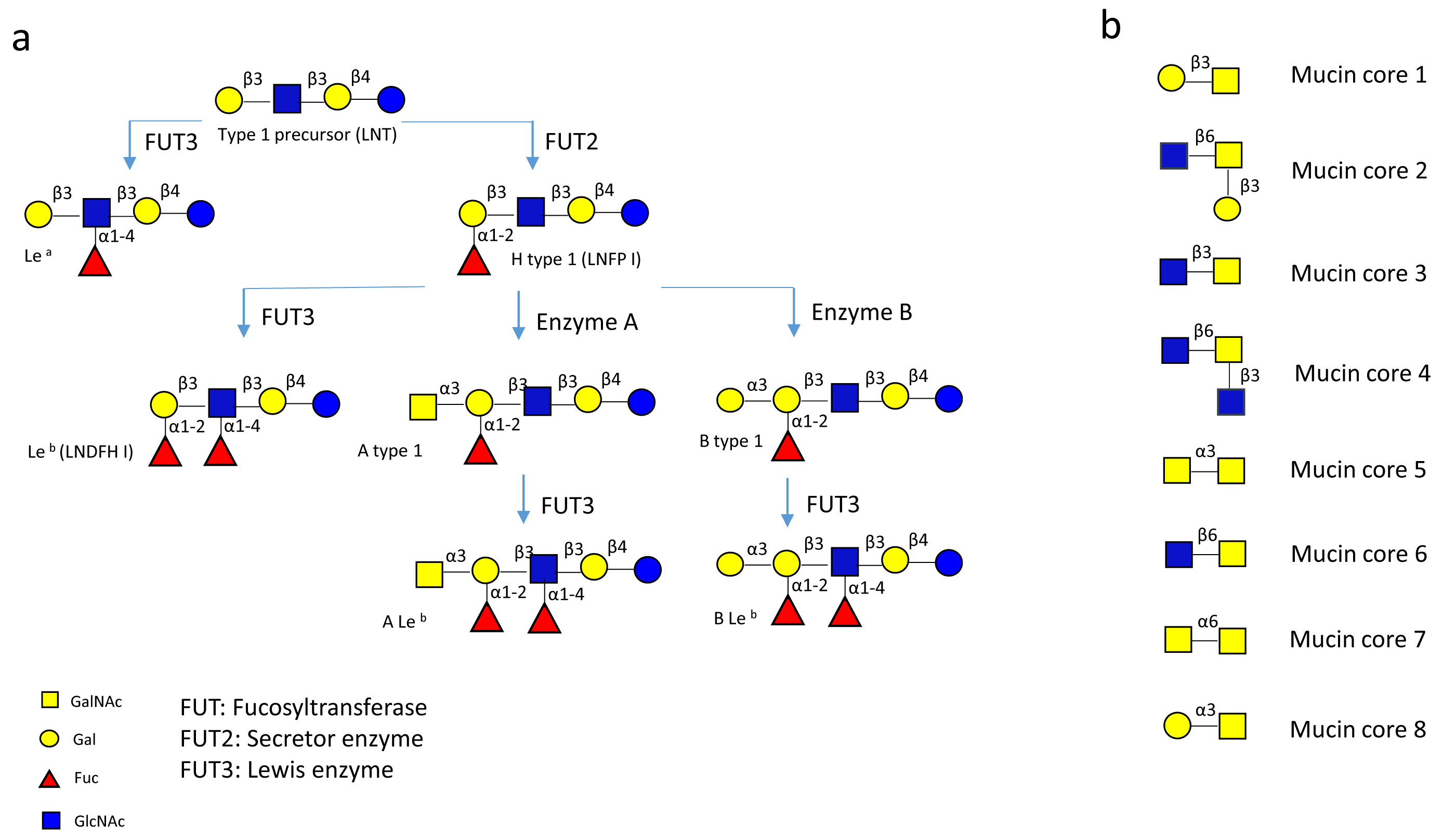

Supplement: S5 Fig — Synthetic pathway of the type 1 histo-blood group antigens (a) and the basic structures of mucin cores (b). (TIF) [file ppat.1006707.s005.tif]
